# Effects of chlorogenic acid on growth, metabolism, antioxidation, immunity, and intestinal flora of crucian carp (*Carassius auratus*)

**DOI:** 10.3389/fmicb.2022.1084500

**Published:** 2023-01-09

**Authors:** Xuexia Jin, Mengyuan Su, Yunxiang Liang, Yingjun Li

**Affiliations:** ^1^State Key Laboratory of Agricultural Microbiology, College of Life Sciences and Technology, Huazhong Agricultural University, Wuhan, China; ^2^Shenzhen Institute of Nutrition and Health, Huazhong Agricultural University, Shenzhen, Guangdong, China; ^3^Shenzhen Branch, Guangdong Laboratory for Lingnan Modern Agriculture, Genome Analysis Laboratory of the Ministry of Agriculture and Rural Affairs, Agricultural Genomics Institute at Shenzhen, Chinese Academy of Agricultural Sciences, Shenzhen, Guangdong, China

**Keywords:** chlorogenic acid, *Carassius auratus*, metabolism, antioxidation, immunity, intestinal flora

## Abstract

In recent years, with the harm caused by the abuse of antibiotics and the increasing demand for green and healthy food, people gradually began to look for antibiotic alternatives for aquaculture. As a Chinese herbal medicine, leaf extract chlorogenic acid (CGA) of *Eucommia ulmoides Oliver* can improve animal immunity and antioxidant capacity and can improve animal production performance. In this study, crucian carp (*Carassius auratus*) was fed with complete feed containing 200 mg/kg CGA for 60 days to evaluate the antioxidant, immuno-enhancement, and regulation of intestinal microbial activities of CGA. In comparison to the control, the growth performance indexes of CGA-added fish were significantly increased, including final body weight, weight gain rate, and specific growth rate (*P* < 0.01), while the feed conversion rate was significantly decreased (*P* < 0.01). Intestinal digestive enzyme activity significantly increased (*P* < 0.01); the contents of triglyceride in the liver (*P* < 0.01) and muscle (*P* > 0.05) decreased; and the expression of lipid metabolism-related genes in the liver was promoted. Additionally, the non-specific immune enzyme activities of intestinal and liver tissues were increased, but the expression level of the adenylate-activated protein kinase gene involved in energy metabolism was not affected. The antioxidant capacity of intestinal, muscle, and liver tissues was improved. Otherwise, CGA enhanced the relative abundance of intestinal microbes, *Fusobacteria* and *Firmicutes* and degraded the relative abundance of *Proteobacteria*. In general, our data showed that supplementation with CGA in dietary had a positive effect on *Carassius auratus* growth, immunity, and balance of the bacteria in the intestine. Our findings suggest that it is of great significance to develop and use CGA as a natural non-toxic compound in green and eco-friendly feed additives.

## Introduction

Crucian carp (*Carassius auratus*) is an important economical freshwater fish with good meat quality, strong disease resistance, and rich nutrition. Thus, it is deeply welcomed by farmers and consumers (Liu et al., 2022). With the continuous improvement of the aquaculture scale and intensification degree, fish diseases caused by microbial pathogens are increasingly aggravated. It is of great significance to the development and use of green and eco-friendly feed additives with effective preventive effects. Chinese herbal medicine, as a natural and innocuous compound, has been used for replacing antibiotics to prevent and control fish diseases (Santos and Ramos, 2016; Zhang et al., 2022).

*Eucommia ulmoides Oliver* is a unique deciduous tree in China, which is listed as top quality in Shennong Ben Cao Jing. *Eucommia ulmoides*’ bark is a valuable tonic and has been used in traditional Chinese herbal medicine formulations for 2,000 years (Luo et al., 2020). Since modern times, some studies have found that the chemical composition of *Eucommia ulmoides’* leaves is basically of similar efficacy to that of *Eucommia ulmoides*’ bark, and the content of some active ingredients in leaves is much higher than that of bark, such as chlorogenic acid (CGA) (Nakamura et al., 1997). CGA has been shown to have a variety of physiological activities, including antibacterial, antiviral (Abaidullah et al., 2021; Chen et al., 2022), protecting the liver (Zhu et al., 2022), anti-tumor (Yagasaki et al., 2000; Belkaid et al., 2006), lowering blood pressure and blood lipids (Zhao et al., 2012), anti-inflammatory (Xu et al., 2020), antioxidant (Liang and Kitts, 2015), and stimulating the central nervous system (Kumar et al., 2019). Additionally, the role of gut microbiota in the protective effect of CGA on obesity and metabolic endotoxemia was identified in mice (Ye et al., 2021). However, the regulatory effect of CGA on immunity and intestinal microecology of freshwater fish in aquaculture remains to be studied.

In this study, we used crucian carp (*Carassius auratus gibelio*) as a model; physiological and biochemical detection technology, high throughput sequencing, real-time fluorescent quantitative PCR, and histological analysis were used to explore the effects of CGA on crucian carp. Our study showed that feeding CGA had a significant positive effect on the growth, immunity, and gut flora balance of *Carassius auratus*. Our findings provide a reference for the strategy of CGA replacing some chemical drugs and antibiotics to prevent and control fish diseases, which is of great significance to healthy aquaculture and ecological environmental protection.

## Materials and methods

### Experimental design, animals, and diets

The experiment was carried out in fish tanks with 1 m^3^ with identical management including water inputs, daily water exchange rate (∼5%), feed type, and rearing schedule. *Carassius auratus* was the Zhongke 3 *Carassius auratus gibelio*. The larval fish were cultured at a stocking density of ∼60 individuals per tank. All the fish were fed a commercial non-medicated feed (Tongwei Co., Ltd.) for 15 days, two times per day at 9 a.m. and 5 p.m. After a 15-day adaptation, the experimental group was fed a diet supplemented with 200 mg/kg CGA for 60 days (Gao et al., 2019), while the control group was fed an ordinary diet. The CGA extract from *Eucommia ulmoides* leaves came from Jiangsu Lvkee Biotechnology Co., Ltd., (Yangzhou, China). The experiments were conducted with three experimental replications. The experimental animal feed formulation is shown in Table 1.

**TABLE 1:** Composition and nutrient levels of experimental diets in this study.

Ingredients	CGA	Con
Crude protein (≥ %)	32.0	32.0
Crude fat (≥ %)	4.0	4.0
Crude fiber (≤ %)	12.0	12.0
Crude ash content (≤ %)	15.0	15.0
Calcium (%)	0.5–2	0.5–2
total phosphorus (≥ %)	0.8	0.8
Moisture (≤ %)	12.5	12.5
Lysine (≥ %)	1.4	1.4
CGA (mg/kg)	200	0

After the experiment, fish were randomly selected from each tank separately at 4 h after the fish feeding in the morning. The fish dissection and sampling were performed as described previously (Li et al., 2020). The gut, liver, and muscle tissue samples were collected separately for subsequent experimental determination. Part of these tissue samples was directly stored at −80°C, and part of them was stored in an RNA protection solution. The foregut of gut was preserved in a formaldehyde fixation solution.

### Growth performance

More than 10 fish were randomly selected from each tank for weighing before and after the experiment. The average body weight, weight gain rate, and specific growth rate of *Carassius auratus* in each group were calculated before and after the experiment, after a 12 h fast. The growth performance indexes were calculated according to the following formula:


weightgainrate(WGR,%)=(Wt-W0)*100/W;0

specificgrowthratio(SGR,%)=(lnW-tlnW)0*100/t;

feedconversionratio(FCR)=F/(W-tW)0;

In the formula:

*W*_0_—*Carassius auratus* initial average body mass (g/tail)

*W*_*t*_—Average body weight of *Carassius auratus* (g/tail)

*t*—Feeding days (d)

*F*—Average total feed intake per fish (g).

### Assay of digestive enzyme, antioxidant, and immune enzyme indices

The tissue of three fish was isolated on an ice tray and homogenized with cold saline with a weight-to-volume ratio of 1:9. The homogenate was centrifuged at 9,000 × *g* for 10 min at 4°C. The supernatant was collected for measuring the indices. The enzyme activity of lipase (LPS) and α-amylase (AMS) in gut tissue homogenate, the antioxidant indices including superoxide dismutase (SOD), peroxidase (POD), reduced glutathione (GSH), and malondialdehyde (MDA) concentration of gut, muscle, and liver tissues, the TG concentration in muscle and liver, and the non-specific immune enzyme activities including acid phosphatase (ACP), alkaline phosphatase (AKP) in gut and liver tissues were determined with commercially assay kits (Nanjing Jiancheng Bioengineering Institute, Nanjing, China) following the manufacturer’s instructions (Matozzo et al., 2013; Wang et al., 2020; Zhao et al., 2021).

### RNA extraction and gene quantification

The total RNA was extracted from the liver and muscle by Biyuntian’s animal tissue RNA extraction kit (Shanghai, China), following the manufacturer’s instructions. After concentration determination (experimental group RNA concentration was 458.261 ng/μL and control group RNA concentration was 650.192 ng/μL), the RNA was reverse transcribed by the reverse transcription kit to obtain cDNA (Nanjing Novezan Biological Co., Ltd., Nanjing, China) (Sun et al., 2018; Tan et al., 2018).

Real-time quantitative PCR (RT-PCR) was performed to quantify the presence of the 12 genes: PPARα (peroxisome proliferator-activated receptor alpha), ACOX1 (acyl-coenzyme A oxidase 1), HSL (hormone-sensitive lipase), ACC (acetyl-CoA carboxylase), FAS (fatty acid synthase), SREBP-1c (sterol regulatory element-binding proteins-lc), DGAT2 (diacylglycerol acyltransferase 2), ATGL (adipose triglyceride lipase), MAGL (monoacylglycerol lipase), AMPK (AMP-activated protein kinase), and EP1α (translation elongation factor 1 alpha) gene which was a reference in the intestinal contents of fish.

The 24 pairs of specific primers were synthesized by Qingke Biotechnology Co., Ltd., (Wuhan, China) and verified by PCR and electrophoresis (Supplementary Table 1). The primer information is shown in Supplementary Table 1. A 10-μl reaction mixture contained 5 μl of the qPCR mix (without ROX) (Zhuangmeng International Biotechnology, Beijing, China), 0.2 μl each of forward and reverse primers (10 μM), 1 μL of template cDNA, and 3.6 μL of DEPC water. Real-time PCR reaction conditions were as follows: the cDNA unchained element was 95°C for 20 s, the denaturation condition was 95°C for 15 s, the annealing condition was 57°C for 20 s, and extension at 72°C for 20 s, for 40 cycles. Fluorescence was read during extension The conditions of the final melting section are 95°C for 15 s, 60°C for 1 min, and 95°C for 15 s. The experiments were conducted with three experimental replications.

### Morphological analysis of intestinal tissue

The morphology of the ileum was analyzed by PAS staining as reported by Wang et al. (2009). Sliced samples were viewed under an optical microscope (Wuhan Sevier Company, Wuhan, China). Five pictures and five fields in each picture were used to analyze villus height, villus number, and goblet cells on villous epithelium using image analysis software under 40 and 200 magnification microscopes (Wang et al., 2009; Yang et al., 2021).

### 16S rRNA gene amplicon sequencing

The genomic DNA of the gut content was extracted using the Fast DNA Stool Mini kit (Qiagen, Valencia, CA, USA) following the manufacturer’s instructions. The primer pair (forward: CCTAYGGGRBGCASCAG and reverse: GGACTACNNGGGTATCTAAT) was used to amplify the V3–V4 region of the 16S rRNA. The obtained amplicons were sequenced on the Illumina Hiseq 2500 platform to generate paired-end 250 bp reads. The resultant paired-end reads were merged using FLASH VI.2.7. The tool QIIME 2 was employed to perform raw read filtration, chimera removal, denoising (with plugin deblur), and taxonomic classification of OTUs (with q2-feature-classifier pre-trained with 99% OTU dataset of the SILVA Release 138). After 30,000 reads per sample (the smallest number of reads per sample) were normalized to generate OTU tables, based on which Chao 1, Observed specie, Shannon, Simpson, and phylogenetic diversity indices were estimated.

### Statistical analysis

All the experimental data were presented as mean ± standard error of the mean (SEM) (Wu et al., 2022). The results were analyzed using a *T*-Test to compare the means of groups. A value of *P* < 0.05 was regarded to be statistically significantly different. Intestinal fecal samples were sequenced by ITS, and OTU clustering was performed according to 97% similarity after quality control, and then compared with UNITE database for annotation (Liang et al., 2020). The 2^–ΔΔ*CT*^ method was used to calculate the mRNA level of target genes accordingly (Livak and Schmittgen, 2001). After OUTs were normalized by the copy numbers, functional pathways were predicted by the Kyoto Encyclopedia of Genes and Genomes (KEGG) catalog at level 3 KEGG orthology groups (KOs) using Phylogenetic Investigation of Communities by Reconstruction of Unobserved States (PICRUSt).

## Results

### Effects of CGA on growth performance of *Carassius auratus*

The effects of oral CGA on the growth performance of crucian carp are shown in Table 2. The fish dietary CGA-added significantly increased the weight, weight gain rate (WGR, *P* = 0.000252) and specific growth rate (SGR, *P* = 0.000305) and tended to reduce the feed coefficient (FCR, *P* = 0.000323) significantly, indicating that the CGA in the diet helps to improve the feed utilization rate and promote the growth of *Carassius auratus*.

**TABLE 2:** Effects of chlorogenic acid on growth performance of *Carassius auratus*.

	The initial weight (g)	Final weight (g)	Weight gain rate (WGR, %)	Specific growth rate (SGR, %)	Feed coefficient (FCR, %)
CGA	7.02 ± 0.33	20.23 ± 0.96**	188.38 ± 3.72**	1.77 ± 0.02**	2.05 ± 0.10**
Con	6.93 ± 0.31	16.47 ± 0.42	137.80 ± 6.08	1.44 ± 0.04	2.83 ± 0.06

**Indicates a significant difference compared with the control group (*P*< 0.01). Data were collected from three independent experiments and are presented as the mean ± SEM. The acronym for the CGA-added group is CGA and the acronym for the control group is Con.

### CGA improves the intestinal digestive enzyme activity of *Carassius auratus*

**FIGURE 1:**
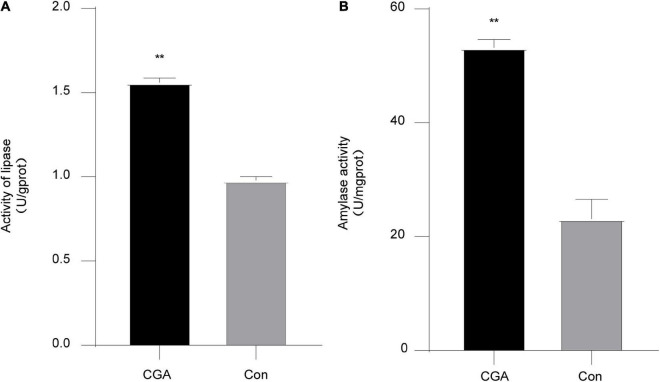
Intestinal digestive enzyme activities. **(A)** Effect of chlorogenic acid (CGA) on intestinal lipase. **(B)** Effect of CGA on intestinal amylase. **Indicates a significant difference compared with the control group (*P*< 0.01). The acronym for the CGA-added group is CGA and the acronym for the control group is Con.

In our study, the intestinal lipase and amylase activities of the CGA-added group were significantly higher than those of the control group, *P* = 0.00007 and *P* = 0.0012, respectively (Figure 1), indicating that adding CGA to the feed could improve the ability of secretion of intestinal lipase and amylase, and enhance the digestion level of fat and starch in *Carassius auratus*.

### Effect of CGA on lipid metabolism and energy metabolism of *Carassius auratus*

**FIGURE 2:**
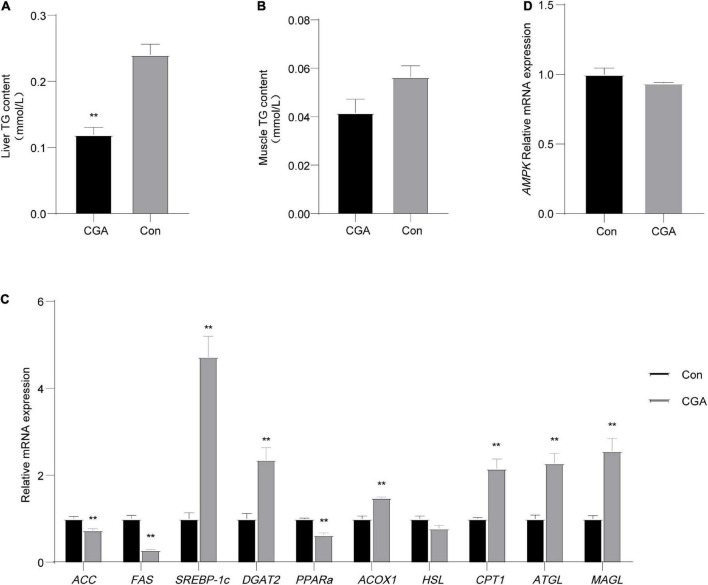
Effect of chlorogenic acid (CGA) on lipid and energy metabolism of *Carassius auratus*. **(A)** Effect of CGA on triglycerides (TG) in the liver. **(B)** Effect of CGA on TG in muscle. **(C)** Expression of lipid metabolism-related genes in liver tissue. **(D)** Expression of energy metabolism gene AMP-activated protein kinase (AMPK) in liver tissue. **Indicates a significant difference compared with the control group (*P*< 0.01). The acronym for the CGA-added group is CGA and the acronym for the control group is Con.

To explore the effect of CGA on the lipid metabolism of *Carassius auratus*, the contents of triglycerides (TG) in the muscle and liver of *Carassius auratus* were determined. As shown in Figure 2A, the TG content in the liver of the CGA-added group was significantly lower than that of the control group (*P* = 0.003), while there had no significant effect on TG content in the muscle (*P* = 0.113) (Figure 2B). In general, CGA had a certain reduction effect on the TG content in the muscle and liver of *Carassius auratus*.

Then we measured the expression of 10 genes related to lipid metabolism in liver tissue. Among these genes, the acetyl-CoA carboxylase (*ACC*) gene, fatty acid synthase (*FAS*) gene, sterol regulatory element-binding proteins-lc (*SREBP-1c*) gene, and diacylglycerol acyltransferase 2 (*DGAT2*) gene were related to fat synthesis, and peroxisome proliferator-activated receptor alpha (*PPAR*α) gene, acyl-coenzyme A oxidase 1 (*ACOX1*) gene, hormone-sensitive lipase (*HSL*) gene, carnitine palmitoyltransferase-1 (*CPT1*) gene, adipose triglyceride lipase (*ATGL*) gene, and monoacylglycerol lipase (*MAGL*) gene were related to lipolysis. As shown in Figure 2C, the expression levels of lipolysis genes (*ATGL*, *CPT1*, *ACOX1*, and *MAGL*) in the CGA-added group were significantly increased (*P* = 0.0065, 0.0064, 0.0016, and 0.0066 respectively), while fat synthesis *gene ACC* and *FAS* in the CGA-added group were significantly decreased (*P* = 0.013 and 0.00089), which may be due to the body self-regulation caused by the less fat content in the body. In general, CGA inhibited the expression of genes related to fat synthesis and promoted the expression of genes related to fat decomposition. Thereby, it is speculated that dietary supplementation of CGA could reduce the storage of fat in the liver, corresponding to the lower hepatic TG content in the CGA-added group than in the control group.

Meanwhile, there was no significant difference in the expression of the AMP-activated protein kinase (AMPK) gene between the CGA-added group and the control group in liver tissue (Figure 2D).

### Non-specific immune enzyme activity in intestinal and liver tissues

**FIGURE 3:**
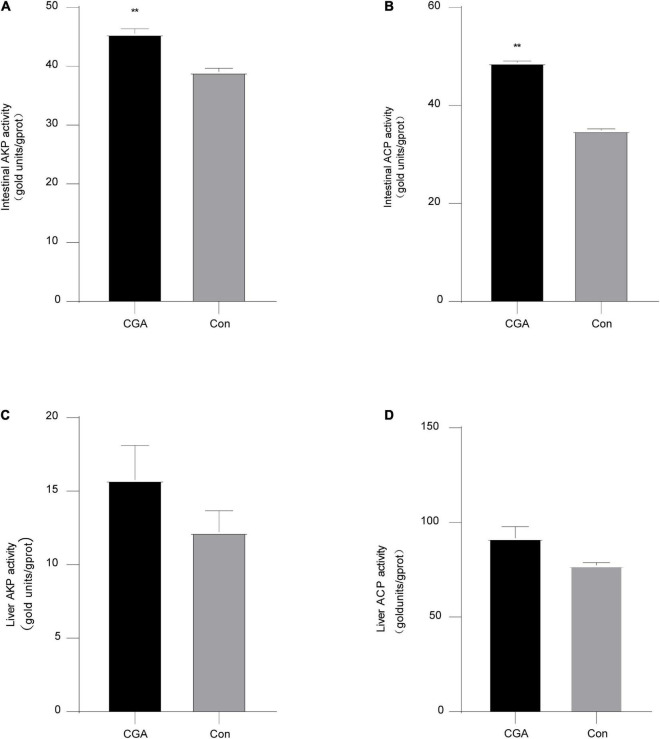
Intestinal and liver non-specific immune enzyme activities. **(A)** Alkaline phosphatase (AKP) activity in intestinal tissue. **(B)** Acid phosphatase (ACP) activity in intestinal tissue. **(C)** AKP activity in liver tissue. **(D)** ACP activity in liver tissue. **Indicates a significant difference compared with the control group (*P*< 0.01). Acid phosphatase (ACP), alkaline phosphatase (AKP). The acronym for the chlorogenic acid (CGA)-added group is CGA and the acronym for the control group is Con.

The non-specific immune responses of fish are related to the enzyme activities of acid phosphatase (ACP) and alkaline phosphatase (AKP). Compared with the control group, ACP (*P* = 0.000006) and AKP (*P* = 0.003627) activities in the CGA-added group were significantly increased in the intestinal tissue (*P* < 0.01). The enzyme activity also increased in the liver tissue, while there was no significant effect (Figure 3). In general, it showed that CGA could improve the liver and intestinal non-specific immunity of *Carassius auratus*.

### Morphological changes of intestinal tissue and the number of goblet cells

**FIGURE 4:**
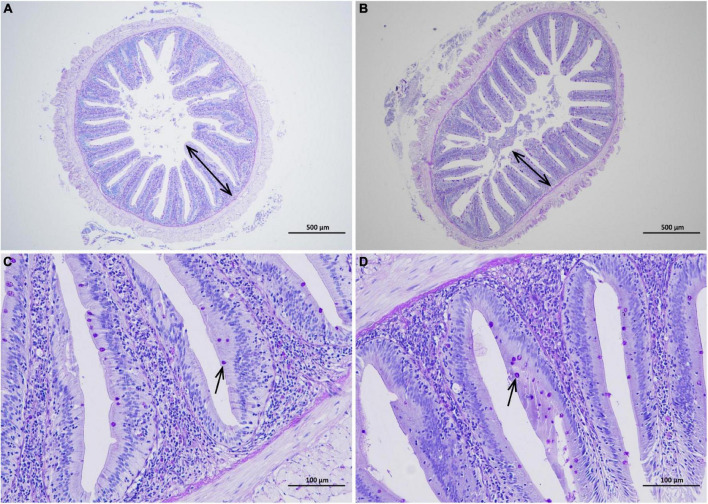
Effects of chlorogenic acid (CGA) on the intestinal morphology. **(A)** Observation of intestinal morphology in the control group under a 40 magnification microscope. **(B)** Observation of intestinal morphology in the experimental group under a 40 magnification microscope. **(C)** Observation of intestinal morphology in the control group under a 200 magnification microscope. **(D)** Observation of intestinal morphology in the experimental group under a 200 magnification microscope. Arrows in panels **(A,B)** indicate villus height. Arrows in panels **(C,D)** indicate goblet cells.

The increase of intestinal villus height and density can cause the extension of the contact area with nutrients and promote nutrient absorption. The mucus layer formed by glycoproteins secreted by goblet cells and immune molecules (such as antimicrobial peptides and cytokines) is the first line of defense against the invasion of pathogenic substances (Alesci et al., 2022). As shown in Figures 4A,B, intestinal villi were leaf-like. Intestinal villi in the CGA-added group were arranged orderly and closely, while sparse and shorter in the control group. Meanwhile, there were slightly more goblet cells in the CGA-added group than in the control group (Figures 4C,D). According to Table 3, dietary supplementation of CGA increased intestinal villus height, villus density, and goblet cells per unit length.

**TABLE 3:** Effect of chlorogenic acid on intestinal morphology of *Carassius auratus.*

	Villus density (n/mm^2^)	Villus height (mm)	Number of goblet cells per unit length (n/mm)
CGA	9.47 ± 1.46*	0.46 ± 0.01	19.46 ± 4.43
Con	12.90 ± 0.89	0.37 ± 0.14	13.57 ± 2.62

*Indicates a significant difference compared to the control group (*P*< 0.05). The acronym for the CGA-added group is CGA and the acronym for the control group is Con.

### Effect of CGA on antioxidant capacity of intestinal tissue, muscle tissue, and liver tissue of *Carassius auratus*

**FIGURE 5:**
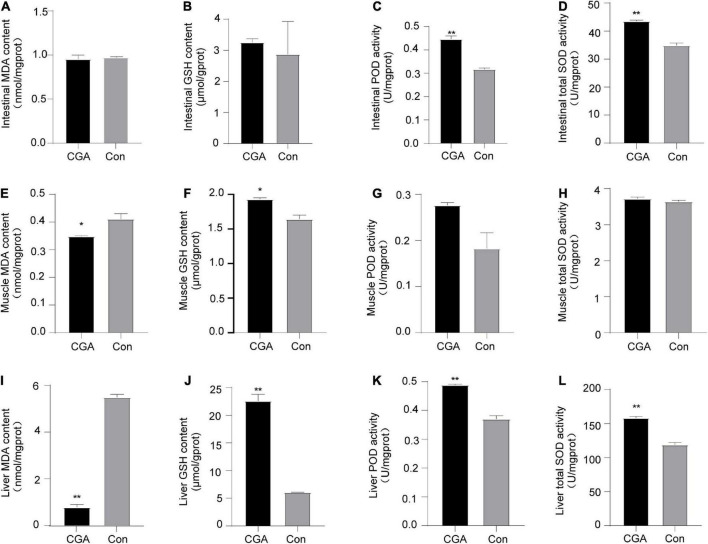
Effect of chlorogenic acid (CGA) on antioxidant capacity of intestinal, muscle, and liver of *Carassius auratus*. **(A)** Malondialdehyde (MDA) content in intestinal tissue. **(B)** Glutathione (GSH) activity in intestinal tissue. **(C)** Peroxidase (POD) content in intestinal tissue. **(D)** Superoxide dismutase (SOD) activity in intestinal tissue. **(E)** MDA content in muscle tissue. **(F)** GSH activity in muscle tissue. **(G)** POD content in muscle tissue. **(H)** SOD activity in muscle tissue. **(I)** MDA content in liver tissue. **(J)** GSH activity in liver tissue. **(K)** POD content in liver tissue. **(L)** SOD activity in liver tissue. Malondialdehyde (MDA), glutathione (GSH), peroxidase (POD), and superoxide dismutase (SOD). *Indicates a significant difference compared to the control group (*P*< 0.05); **Indicates a significant difference compared with the control group (*P*< 0.01). The acronym for the CGA-added group is CGA and the acronym for the control group is Con.

Lipid peroxidation can cause the formation of MDA, which is an indicator of the degree of oxygen-free radical damage in the human body. In our study, we found that when CGA was added to the fish diet, MDA content in multiple tissues was reduced (Figures 5A,E,I). Especially, the content of MDA in the liver tissue was significantly lower than that in the control group (*P* = 0.000012). Three antioxidant enzymes, namely GSH, POD, and SOD, are important participants in cellular anti-oxidation. Compared with the control group, the antioxidant GSH in intestinal, muscle, and liver tissue of the CGA-added group were all increased (Figures 5B,F,J). Additionally, the activities of POD and total SOD in the CGA-added group were higher than those in the control group (Figures 5C,D,G,H,K,L).

It is worth noting that the MDA, GSH, POD, and SOD in the CGA-administrated liver tissue of *Carassius auratus* were all significantly different from those in the control group. These results showed that supplementation of CGA in diet could improve the antioxidant capacity of *Carassius auratus*, especially in the liver.

### Analysis of intestinal microbial diversity

An Illumina Hiseq 2500 sequencing platform was utilized to analyze the structure of the fish gut microbiota. A total of 499,848 microbial 16S rRNA genes raw reads were assembled using FLASH at quality settings, obtaining 492,686 clean reads. After applying the Usearch clustering algorithm, all 2,947 unique OTUs were identified and allotted.

**FIGURE 6:**
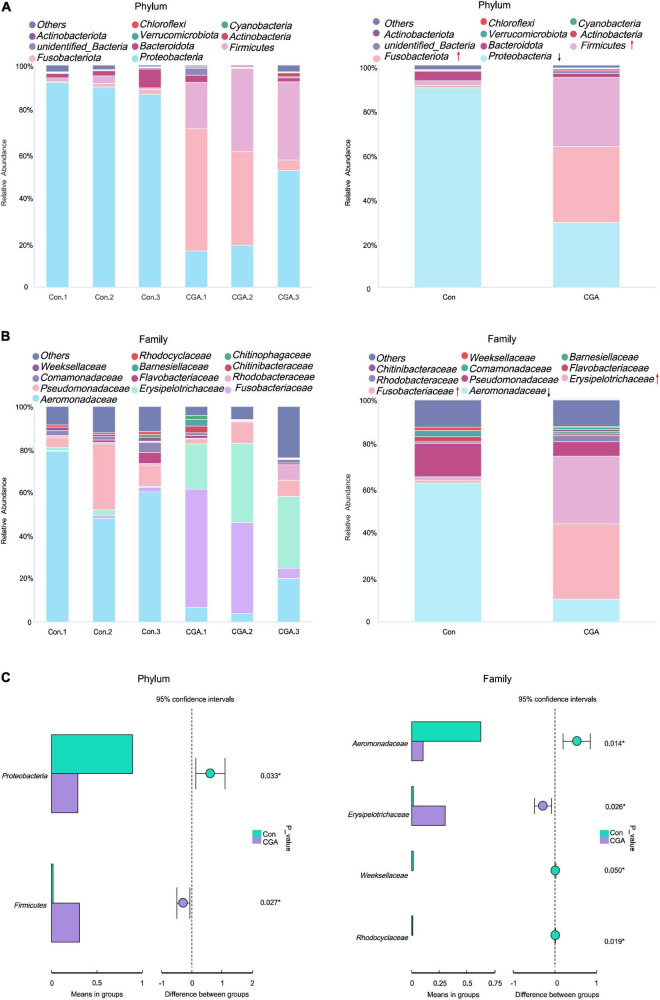
Bar chart of relative abundance of intestinal species. **(A)** Histogram of phyla level relative species abundance. **(B)** Histogram of relative abundance of species at the family level. **(C)** The changes of the main species between the two groups. con.1, con.2, and con.3 are the control group (Con), and chlorogenic acid (CGA).1, CGA.2, and CGA.3 are the CGA-added group (CGA). The green box represented Con; the purple box represents CGA. *Indicates a significant difference compared to the control group (*P*< 0.05).

Furthermore, the core microbial communities in each group were analyzed at the phylum and family levels (Figures 6A,B). Four predominant phyla accounted for more than 96% abundances of the total sequences at the phylum level, including *Proteobacteria*, *Fusobacteriota*, *Firmicutes*, and *Bacteroidota* in both two groups (Figure 6A). The relative abundance of *Proteobacteria* occupied the vast majority in the control group, and supplementation of CGA in the fish diet significantly increased the abundance of *Firmicutes* (from 2.00 to 31.27%, *p* = 0.027) and decreased the abundance of *Proteobacteria* (from 89.95 to 29.29%, *p* = 0.033) (Figure 6C).

The bacteria with higher abundance (> 0.01%) was shown in Figure 6B. The families *Aeromonadaceae* and *Pseudomonadaceae* dominated in the control group, and their relative abundances were 62.74 and 15.01%, respectively (Figure 6B). In the CGA-added group, The families *Fusobacteriaceae* and *Erysipelotrichaceae* were predominant, and their relative abundances were 34.10 and 30.40%, respectively. Otherwise, the relative abundance of the *Aeromonadaceae* significantly decreased from 62.74 to 10.27% as the CGA was added (Figure 6C). In summary, the composition of dominant bacterial communities was affected by dietary supplementation of CGA.

The dynamics of alpha diversity were further studied. As shown in Table 4, the Ace, Chao1, and observed species indices reduced from 148 to 142, 150 to 144, and 146 to 138, respectively, as CGA was added to the feed. Meanwhile, Shannon and Simpson’s indices increased from 3.05 to 3.22 and 0.72 to 0.79, respectively. Accordingly, the alpha diversity showed a slightly declined trend with the supplementation of CGA in the fish diet.

**TABLE 4:** Effect of chlorogenic acid on alpha diversity index of intestinal bacteria.

	Ace	Chao1	Observed species	Shannon	Simpson
Exp	142 ± 19	144 ± 18	138 ± 23	3.22 ± 0.49	0.79 ± 0.05
Con	148 ± 6	150 ± 7	146 ± 7	3.05 ± 0.52	0.72 ± 0.12

Ace and Chao1 are the flora abundance index, observed species are the sequencing depth index, and Shannon and Simpson are the flora diversity index. The acronym for the CGA-added group is CGA and the acronym for the control group is Con.

### 16S rRNA functional prediction analysis

**FIGURE 7:**
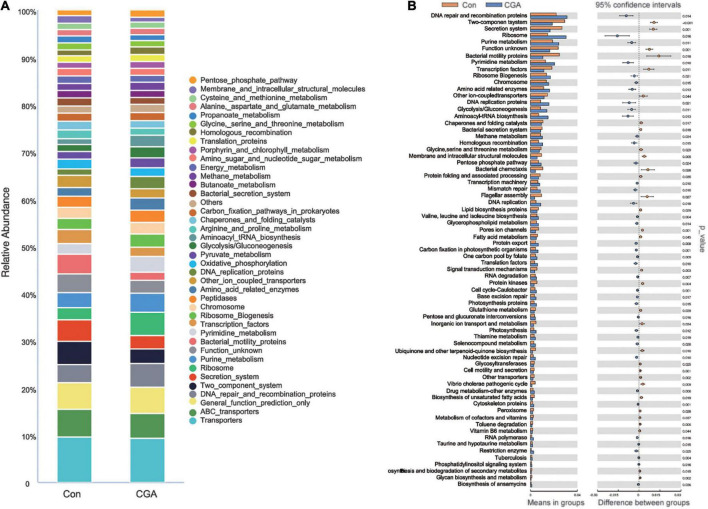
Kyoto Encyclopedia of Genes and Genomes (KEGG) pathways analysis by Phylogenetic Investigation of Communities by Reconstruction of Unobserved States (PICRUSt). **(A)** Relative abundance of KEGG pathways for two groups. **(B)** KEGG pathways with significant differences between two groups (*T*-test). The acronym for the chlorogenic acid (CGA)-added group is CGA and the acronym for the control group is Con.

To analyze the functions of gut microbiota among the samples, sequences were conducted and submitted to the KEGG database for analysis. Metagenome potentials were predicted by PICRUSt. The top 40 relative abundance of KEGG pathways are revealed in Figure 7A, and the KEGG pathways with significant differences with supplementation of CGA in fish diet are shown in Figure 7B. Briefly, the largest relative abundance of KEGG pathways was found in transporters, general function prediction, and the DNA repair and recombination proteins both in the two groups. The relative abundance of amino acid-related enzymes, DNA repair and recombination proteins, and DNA replication was significantly increased with supplementation of CGA in fish diet.

## Discussion

Our study indicated that the feeding supplemented with 200 mg/kg CGA had a positive effect on the growth performance of *Carassius auratus*. It has been reported that CGA can help to promote piglet growth and improve the feed conversion ratio (Chen et al., 2018). The mechanism of action may be related to the significant increase of the intestinal amylase and lipase activities of *Carassius auratus*, as the enhancement of the digestive function of the intestine will contribute to the full absorption of nutrients. Meanwhile, oral CGA also had a positive effect on the density and height of the intestinal villi of *Carassius auratus*. The density and height of villi in the intestine can increase the contact area between the intestine and intestinal contents, thus increasing the deposition of nutrients, which is of great significance for improving the health of animals (Fu et al., 2021).

Acid phosphatase and AKP are important hydrolases and play an important role in the body’s immune defenses (Faccioli et al., 2016). These two enzymes, collectively called phosphatase, are divided according to the optimal PH value of catalytic conditions. They can participate in the process of protein dephosphorylation, digestion, and absorption of nutrients and other functions, and are also an important detoxification system in the body. In our study, CGA significantly increased the activities of ACP and AKP in the intestinal tissue of *Carassius auratus*, that corresponded to the results of the increase in the number of goblet cells per unit length, which both improved intestinal immunity to a certain extent. Additionally, TG content in liver tissue decreased significantly as fed with CGA, which corresponded to the result of the expression of genes related to lipid metabolism in liver tissue. These results indicated that feeding supplemented with CGA could promote lipid metabolism in the liver of *Carassius auratus*, thus reducing the accumulation of lipids in liver tissue.

The increase of free radical content in organisms may lead to an increase in lipid peroxidation content and lipid peroxidation damage, and the decomposition of lipid peroxides will produce some harmful substances, such as MDA (Ayala et al., 2014). Therefore, the key to maintaining the health of the body is to eliminate the free radical content of the body, inhibit the appearance of lipid peroxidation damage, and protect the integrity and function of the cell membrane. The antioxidant enzymes total SOD, POD, and some non-enzymatic antioxidant substances such as GSH can eliminate the reactive oxygen free radicals of the body, thus improving the antioxidant capacity of the body (Liu et al., 2013). We found that feeding CGA could reduce the production of lipid peroxides in the *Carassius auratus* intestinal and muscle, and improve the total antioxidant enzyme SOD, POD, and the antioxidant substances GSH levels of the liver tissue. Thus, the enhancement of the metabolism index inflected the improvement body’s antioxidant and immune capacity. In our study, there was no significant difference in the expression of the AMP-activated protein kinase (AMPK) gene between the CGA-added group and the control group in liver tissue (Figure 2D). The results of Kong et al. show that under the action of CGA, the expression of AMPK was significantly increased, the expression of FAS was decreased, and the expression of HSL was increased (Kong et al., 2021), which was partially consistent with the results of this study, and it may be caused by the different growth cycles of the experimental crucian carp.

Intestinal microorganisms play an important role in the growth and development of fish as well as their immune ability. It was reported that CGA-induced changes in the gut microbiota played an important role in the inhibition of metabolic endotoxemia in mice (Ye et al., 2021). In this study, a change in *Carassius auratus*’ gut microbial composition was found as the application of CGA. According to previous studies, *Proteobacteria* and *Firmicutes* were the dominant phyla in the intestinal tract of fish (Zou et al., 2020). It was reported that the gut microbiota of *Carassius auratus* was initially dominated by *Proteobacteria*, and in adulthood, it was jointly dominated by *Proteobacteria*, *Fusobacteriota*, and *Firmicutes* (Li et al., 2017). Additionally, these three phyla play an important role in the growth and metabolism of fish. *Proteobacteria* have been reported to be involved in the metabolism and cycling of carbon, nitrogen, and sulfur in fish (Fjellheim et al., 2010; Han et al., 2010); *Bacteroidota* is involved in the fermentation process and degradation of oligosaccharides (Li et al., 2015). *Firmicutes* contribute to carbon metabolism (Corrigan et al., 2015). In this study, our results were similar to those reported. In the control group, *Proteobacteria* was dominated by intestinal bacteria and the relative abundance of *Proteobacteria* was 91.76%, *Fusobacteriota* was 1.33%, *Firmicutes* was 1.68%, and *Bacteroidota* was 4.21%. While in the CGA-added group, fish gut microbes mainly composed by *Proteobacteria*, *Fusobacteriotas*, and *Firmicutes*. Practically, *Proteobacteria* accounted for 30.13%, *Fusobacteriota* 34.52%, *Firmicutes* 31.50%, and *Bacteroidota* 1.48%. The relative abundance ratio of *Firmicutes* and *Bacteroidota* may also be related to the growth of the fish (Li et al., 2013).

Chlorogenic acid had various antimicrobial effects on CGA, while it was not sensitive to probiotic bacteria which made it even more appropriate to use in the food industry (Naveed et al., 2018). In our study, the relative abundance of *Aeromonadaceae*, *Fusobacteriaceae*, *Erysipelotrichaceae*, and *Pseudomonadaceae* was increased in the intestinal bacteria of *Carassius auratus* with the application of CGA. *Aeromonadaceae* and *Pseudomonadaceae* are essential colony members in the normal intestinal bacteria of fish and are potential probiotics that play an important role in the digestion and health of fish (Wu et al., 2012). Additionally, it was reported that the *Erysipelotrichaceae* family could help to digest high-fat and polysaccharide diets. Meanwhile, *Erysipelotrichaceae* may enhance fish immunity by stimulating the TLR4 pathway, which was involved in innate immune defense in teleost fish (Conterno et al., 2011; El Kasmi et al., 2013; Harris et al., 2014; Qi et al., 2017). Some strains of the *Fusobacteriaceae*, such as *Cetobacterium somerae*, played a catalytic role in the synthesis of vitamin B12 in fish (Tsuchiya et al., 2008). Therefore, the increased relative abundance of *Aeromonadaceae*, *Fusobacteriaceae*, *Erysipelotrichaceae*, and *Pseudomonadaceae* in the intestinal bacteria may promote the immunity and growth performance of *Carassius auratus* as the application of CGA. The composition and diversity of intestinal flora can predict different biological functions. In this study, KEGG analysis revealed that one of the most abundant KOs related to DNA repair, recombination, and replication was significantly increased with supplementation of CGA in a fish diet, which showed that CGA may have anti-inflammatory and antioxidant physiological activities.

## Conclusion

This study revealed that the growth performance of *Carassius auratus* fed with CGA was significantly improved which may be related to the enhancement of the intestinal digestive enzyme activity and increased density and height of the intestinal villi. It was shown that CGA is of great significance for improving the growth of animals. In addition, our results showed that feeding supplemented with CGA could increase the expression of genes related to lipid metabolism in the liver, improve the activities of non-specific immune enzymes in intestinal and liver tissues, promote the antioxidant capacity of intestinal, muscle, and liver tissues. Meanwhile, our data also demonstrated the relative abundance of the most dominant bacterial communities interchangeably with dietary supplementation of CGA. At the phylum level, *Proteobacteria* decreased and *Firmicutes* increased in the CGA-added group. At the family level, *Fusobacteriaceae* and *Erysipelotrichaceae* were the dominant families in the CGA-added group, while the relative abundance of *Aeromonadaceae* decreased significantly.

Overall, our study showed that supplementation with CGA in diet had a significant positive impact on *Carassius auratus*. It is of great significance to develop and use CGA, a natural and innocuous compound, in green and eco-friendly feed additives with effective preventive effects. Our findings provided a reference for exploring the CGA for replacing some chemical drugs and antibiotics to prevent and control fish diseases.

## Data availability statement

The datasets presented in this study can be found in online repositories. The names of the repository/repositories and accession number(s) can be found below: https://www.ncbi.nlm.nih.gov/, PRJNA897832.

## Ethics statement

The animal study was reviewed and approved by the university’s Animal Care and Use Committee (SCXK2020-0084) and performed in accordance with internationally accepted guidelines and ethical principles.

## Author contributions

XJ and MS: designed research ideas and content, wrote the manuscript, performed experiments, and analyzed data. YXL and YJL: project administration and funding acquisition. YJL: reviewed and edited the manuscript. All authors contributed to the article and approved the submitted version.
