# Supplementary material for: Effects of chlorogenic acid on growth, metabolism, antioxidation, immunity, and intestinal flora of crucian carp (Carassius auratus)
Source: Front Microbiol. 2023 Jan 9;13:1084500. doi: 10.3389/fmicb.2022.1084500 (PMC9868665; doi:10.3389/fmicb.2022.1084500)
Supplement: Supplementary file 1 [file Table_1.DOCX]

### Supplemental files

### Effects of chlorogenic acid on growth, metabolism, antioxidation, immunity and intestinal flora of Crucian carp (*Carassius auratus*)

**Xuexia Jin^1#^, Mengyuan Su^1#^, Yunxiang Liang** **^1*^ and Yingjun Li^1, 2, 3, 4*^**

1 *State Key Laboratory of Agricultural Microbiology and* *College of Life Science and*

*Technology,* *Huazhong Agricultural University, 430070 Wuhan, China*

2 *Shenzhen Institute of Nutrition and Health, Huazhong Agricultural University, Shenzhen, Guangdong 518000, P. R. China*

3 *Shenzhen Branch, Guangdong Laboratory for Lingnan Modern Agriculture, Genome Analysis Laboratory of the Ministry of Agriculture and Rural Affairs, Agricultural Genomics Institute at Shenzhen, Chinese Academy of Agricultural Sciences, Shenzhen, Guangdong 518120, P. R. China*

4 *College of Biological and Environmental Engineering, Xi'an University, 710065 Xi'an, P. R. China*

*** Correspondence:** Yingjun Li, [yingjun@mail.hzau.edu.cn](mailto:yingjun@mail.hzau.edu.cn); Yunxiang Liang, [fa-lyx@163.com](mailto:fa-lyx@163.com)

# These authors contributed equaly to this work.

Table S1. Gene cloning primer information

| Primer name | Sequence (left to right 5 'to 3') | bases |
| --- | --- | --- |
| CPT1-F(liver) | ACCTCCAGCTCAGTCATGAGG | 21 |
| CPT1-R(liver) | GACGAACAGGCCCAAGGAAG | 20 |
| ACC-F | ACCCACTGGCAAACAGATCC | 20 |
| ACC-R | GTCTTTGTGCCCCACCTGTC | 20 |
| FAS-F | GACTCGGAAGGGCGAGAGAG | 20 |
| FAS-R | CTTTGATTCCGGATGTGGGTACAG | 24 |
| SREBP-F | CACCCTCCACGACTGTCAAC | 20 |
| SREBP-R | CGGTCGTGGATGATACCGAG | 20 |
| DGAT2-F | GCACAACCAACGAAGTTATTGCC | 23 |
| DGAT2-R | TGCTTCACCTCCACTCTTTGG | 21 |
| ATGL-F | ACTGTGGCCTGATACCTCCAAC | 22 |
| ATGL-R | ATGTCTCGAGGGCAGATGTCAC | 22 |
| MAGL-F | GATTCAGACTCAGCCCTCGG | 20 |
| MAGL-R | CCATGGGCAACAAACACCAG | 20 |
| AMPK-F | TCGCAATCTCCAGGATCCTCTTGC | 24 |
| AMPK-R | GTGAATCTTGGCAGAGTGGTGCG | 23 |
| EP1α-F | CGTGTCTGTCAAGGACATCCGC | 22 |
| EP1α-R | CTGACCAGGGTGGTTCAGGATG | 22 |
| PPARα-F | TGGACCTCAACGACCAGGTG | 20 |
| PPARα-R | CAGAAATTCTCGGGTGATGAAGCC | 24 |
| ACOX1-F | GGAGCTACAGCACAGCAAGAG | 21 |
| ACOX1-R | GCATAAAGCAGAGCCAAAGTGC | 22 |
| HSL-F | TCTTCCACTGTGGTGCAAGTG | 21 |
| HSL-R | TGTCCTGTCCTTGTCGGAGTG | 21 |

Note: Primer name - before is gene abbreviation, F is forward primer, R is reverse primer.
